# Cost-Effectiveness of Vitamin D Supplementation in Pregnant Woman and Young Children in Preventing Rickets: A Modeling Study

**DOI:** 10.3389/fpubh.2020.00439

**Published:** 2020-09-04

**Authors:** Vilius Floreskul, Fatema Z. Juma, Anjali B. Daniel, Imran Zamir, Andrew Rawdin, Matthew Stevenson, Zulf Mughal, Raja Padidela

**Affiliations:** ^1^School of Health and Related Research, The University of Sheffield, Sheffield, United Kingdom; ^2^Dolon Ltd, London, United Kingdom; ^3^Faculty of Biology, Medicine and Health, Manchester Academic Health Science Centre, University of Manchester, Manchester, United Kingdom; ^4^Royal Manchester Children's Hospital, Manchester University NHS Foundation Trust, Manchester, United Kingdom; ^5^North Manchester General Hospital, Manchester, United Kingdom

**Keywords:** rickets, prevention, cost-effectiveness, incremental cost-effectiveness ratio, quality adjusted life years, decision tree model

## Abstract

**Background:** Literature on the cost of management of rickets and cost-effectiveness of vitamin D supplementation in preventing rickets is lacking.

**Methods:** This study considered the cost-effectiveness of providing free vitamin D supplementation to pregnant women and children <4 years of age with varying degrees of skin pigmentation to prevent rickets in children. Estimates for the prevalence of rickets were calculated using all cases of rickets diagnosed in Central Manchester, UK and census data from the region. Cost of management of rickets were calculated using National Health Service, UK tariffs. The efficacy of vitamin D supplementation was based on a similar programme implemented in Birmingham. Quality of life was assessed using utility estimates derived from a systematic literature review. In this analysis the intervention was considered cost-effective if the incremental cost-effectiveness ratio (ICER) is below the National Institute for Health and Care Excellence, UK cost-effectiveness threshold of £20,000 per Quality-adjusted life year (QALY).

**Results:** Fifty-seven patients (26 dark, 29 medium and 2 light skin tones) were managed for rickets and associated complications over 4-years. Rickets has an estimated annual incidence of 29·75 per 100,000 children <4 years of age. In the dark skin tone population vitamin D supplementation proved to be cost saving. In a medium skin tone population and light skin tone populations the ICER was £19,295 per QALY and £404,047 per QALY, respectively.

**Conclusion:** Our study demonstrates that a vitamin D supplementation to prevent rickets is cost effective in dark and medium skin tone populations.

**Research in Context**

**Evidence Before This Study**

Literature on the cost of management of rickets and cost-effectiveness of Vitamin D supplementation in preventing rickets is lacking in the UK and worldwide. Global consensus guidelines on Vitamin D rickets noted the lack of data to provide guidance on cost-effectiveness of supplementation (1).

**Added Value of This Study**

This study investigated the cost-effectiveness of an intervention where vitamin D supplements would be provided free of charge to pregnant women and children under 4 years of age in order to prevent rickets. Skin pigmentation is a risk factor for the development of vitamin D deficiency rickets (1) and hence this analysis assesses the intervention's cost-effectiveness in the general population and at-risk subgroups consisting of participants with varying degrees of skin pigmentation. Results from this study shows that the intervention is estimated to be clinically beneficial and cost-saving in participants with medium and dark-skin tone, respectively.

**Implications of All the Available Evidence**

There is evidence to support population-based vitamin D supplementation of pregnant women and children under 4 years of age as it reduces incidence of rickets (2). In this study we present evidence of cost-effectiveness of vitamin D supplementation in pregnant women and children <4 years of age with varying degrees of skin pigmentation in UK. Health care policymakers can use this evidence in implementing population-based interventions to reduce the prevalence of rickets and its complications in at-risk populations.

## Introduction

Vitamin D is a fat-soluble nutrient required for calcium and phosphorus homeostasis and for skeletal mineralization. Vitamin D deficiency as reflected by serum 25-hydroxy vitamin D (25OHD) concentration of <30 nmol/l is associated with musculoskeletal and non-musculoskeletal manifestations (3). Musculoskeletal outcomes of vitamin D deficiency includes rickets and osteomalacia in children, osteomalacia in adults, reduced muscle strength and increased risk of falls (3). Non-musculoskeletal manifestations of vitamin D deficiency include increased risk of cancers, cardiovascular diseases, auto-immune diseases, all-cause mortality, infectious diseases, and age-related macular degeneration (1, 3).

The prevalence of vitamin D deficiency in the general population in England and Wales is 23% (3). Endogenous synthesis of Vitamin D occurs when ultraviolet-B radiation penetrates into the epidermis. Skin pigmentation interferes with sub-cutaneous vitamin D synthesis; as a result, deficiency prevalence is as high as 80% in population with darker skin tones and therefore they are at a higher risk of developing skeletal and extra-skeletal manifestation of Vitamin D deficiency than people with a lighter skin tone (3, 4). In addition deficiency has also been reported in vegans and vegetarians residing in UK (5).

Vitamin D supplementation, is known to be effective in preventing skeletal manifestations of vitamin D deficiency such as rickets and it's associated complications (2). While there is uncertainty about vitamin D deficiency preventing extra-skeletal manifestations, the recent individual patient metanalyses by Martineau and colleagues showed that it was effective in reducing risk of respiratory tract infections, especially in those who are vitamin D deficient (6).

Rickets results in bone pain, impaired growth, dental enamel hypoplasia and skeletal deformities including abnormally shaped skull, bowed legs, knocked knees, chest deformities and swelling of ends of the long bones. In severe cases, deficiency can cause hypocalcaemic seizures and life-threatening dilated cardiomyopathies (3, 7). Rickets and vitamin D deficiency can result in considerable costs to the National Health Service (NHS) especially when compared with the cost of vitamin D supplements, though there is a lack of evidence on this in the UK and worldwide (3). Rickets and vitamin D deficiency are also associated with the possibility of lifetime complications which if unaddressed can result in significant disutility (3).

Currently, the NHS operates the “Healthy Start” scheme for low-income households where multivitamin supplements, including 10 μg (400 IU) of vitamin D, are provided free of charge to pregnant woman and children under 4 years of age, with other families advised to buy the recommended supplements (8). However, uptake of Healthy Start vitamins is very low and cases of rickets are continued to be reported in the UK (9, 10).

Historically a table spoon of cod liver oil (5 ml containing 400 IU of vitamin D) was found to be effective in prevention rickets in children (11, 12). Following extensive review of literature and expert opinion, global consensus guidance recommends 400 IU of vitamin D as an effective dose in preventing rickets (1). In UK, the current DOH recommends 7·0 and 8·5 μg of vitamin D per day and in addition pregnant and breastfeeding women should receive 10·0 μg of vitamin D per day to ensure that the mother does not suffer from vitamin D deficiency thus avoiding neonatal hypovitaminosis (1, 3). To the best of our knowledge the cost-effectiveness of this public health recommendation has not been evaluated. Thus, the purpose of this study was to determine if Healthy Start vitamin D supplementation of all pregnant women and children under 4 years of age or only those with a darker skin tone who are at a higher risk of developing vitamin D deficiency associated rickets would be cost-effective in preventing rickets in children.

## Methods

### Study Perspective

Analysis was conducted from a NHS and personal social services (PSS) perspective, following the National Institute's for Health and Care Excellence (NICE) reference case and was focussed on the population of Central Manchester, UK (13). The objective was to ascertain the cost-effectiveness of vitamin D supplementation in pregnant women and in children <4 years of age in preventing rickets in children in the following skin tone subgroups: light, medium, and dark.

### Target Population

Skin pigmentation is a risk factor for rickets and therefore separate analyses were conducted for population subgroups consisting of pregnant women and children <4 years of age with light, medium and dark skin tones. Skin tone assignments were based on rickets patients' ethnicity. Patients of Afro-Caribbean origin were considered to have dark skin tone; those from South Asian and Middle Eastern origin medium skin tone and those of European origin light skin tone. After analyzing the observational dataset, it was found that 95% of the rickets cases (95% CI 0·86–0·98) had onset before the age of 48 months. For this reason, children under 4 years of age were considered to be the primary target population, which is in line with the existing recommendations. Skin tone specific and overall prevalence of rickets in the target area were estimated using this observational dataset and census data (14).

### Analytical Methods and Conceptual Model

**Figure 1:**
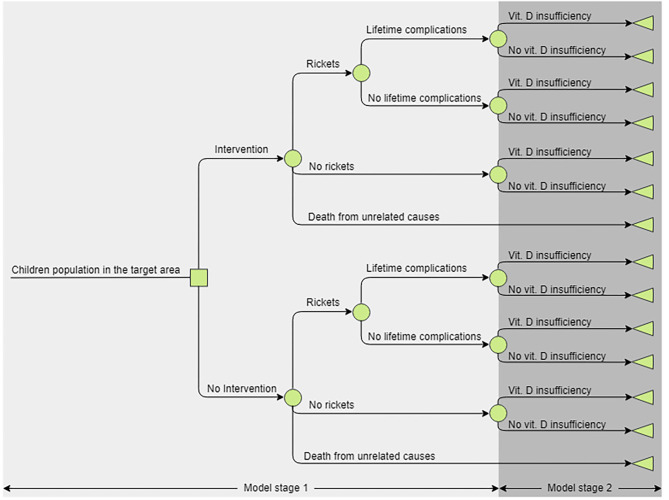
The structure of the decision tree analytic model. The model consists of two arms—current and alternative practices. Stage 1 represents the first 4 years of life, if a child developed rickets they could recover completely or recover but experience lifelong complications, children could also die as a result of all-cause mortality. In Year 5 all alive children transition to stage 2, where they can be vitamin D deficient and in their latter life experience disutility and increased mortality risk, or not. Children would remain in the stage 2 for the remainder of their lifetime as the deficiency prevalence in the cohort is assumed to be constant.

A decision tree model (Figure 1) was developed in Microsoft Excel (^©^Microsoft Corporation). In addition to rickets, vitamin D deficiency is associated with other health outcomes, prevalence of which would change if prevalence of vitamin D deficiency changed (15). To account for this, the model included increases in mortality and disutility associated with vitamin D deficiency (3). They were chosen because evidence did not allow the quantification of causal links for more specific conditions, assuming an increased all-cause mortality summarized the effects of fatal outcomes, e.g., cancer; and non-fatal outcomes were summarized by assuming a disutility.

Vitamin D supplementation during pregnancy decreases the risks of infant vitamin D deficiency rickets (3). The model accounts for this as the observational dataset accounts for infant rickets cases related to their mother's vitamin D deficiency and cases developed post-birth; also intervention effect estimates capture the effect of vitamin D supplementation pre and post birth. Infant formula is enriched with vitamin D and formula fed infants should not be supplemented. The model accounts for this in the same manner as to prenatal supplementation.

### Time Horizon

In the base-case, the time horizon is 4 years, the period over which vitamin D supplements would be provided free of charge. After this period supplementation and vitamin D deficiency rates, would eventually return to baseline with no differences in costs or utilities between the study groups expected during this time. Due to the limited data on lifelong complications, the base-case analysis assumes no incidence of lifelong complications, the impact of these assumptions is investigated in the scenario analysis where the time horizon is increased to lifetime in accordance with the NICE reference case.

### Health Outcomes

Following the NICE reference case an attempt was made to measure health outcomes using quality-adjusted life years (QALYs) (13). To do this utility and disutility estimates were required for the health states included in the model, again according to the NICE reference case these utilities should be measured using a preference based instrument. However, no preference-based instrument is validated for use in early childhood and estimates of HRQoL in the included health states are unknown. Thus, counterfactual utility estimates were derived from age adjusted population baseline and disutility estimates.

Baseline utility was estimated using an UK algorithm for EQ-5D-3L:

$$\begin{array}{l} {\text{U}}_{\text{BASELINE}}=0\cdot 950857-0\cdot 000259{\;}^{*}{\text{ age}}^{2+}0\cdot 021213{\;}^{*}\text{ gender} \end{array}$$

Where gender = 1 for males and 0 for females (16, 17).

Two systematic literature searches were conducted to identify evidence on HRQoL in rickets and vitamin D deficiency. Expert knowledge suggested non-existing evidence on HRQoL in life-long complications and scoping searches were not promising. As a result, systematic literature search to inform this parameter, which is only used in scenario analysis, when the time horizon extends to lifetime, was not conducted and the analysis relied on expert estimate.

Rickets and vitamin D deficiency related HRQoL targeted searches are reported in the Supplementary Appendix. The former resulted in identification of a single study, the latter two studies, one of which was excluded due to population differences (18–20).

The study for HRQoL in rickets was assessed to have a high risk of bias, and hence was later replaced by a higher quality study (Table S1) published after the systematic literature search, identified by experts in the field (21). This study reported utility values derived from proxy valuations using a preference-based HRQoL tool: Child Health Utility 9 Dimension (CHU9D) questionnaire, preference-weighted by the general population in the UK (22). There was no information on the duration of rickets-related disutility, hence expert estimates were used.

The study on vitamin D deficiency-related HRQoL was assessed as being at low risk of bias. HRQoL in this study was measured using EQ-5D-5L and converted to utility values using a US valuation study (23). As the conversion method recommended by the Decision Support Unit (research body commissioned by NICE to provide methodological support) required the scores from the individual dimensions of the EQ-5D-5L to convert to the 3D version, which were not published, proportionate disutility, with reference to the baseline was estimated on the EQ-5D-5L scale and then assumed generalizable to the EQ-3D-3L scale (24). This disutility is only considered in the scenario analysis, when the time horizon extends to lifetime, as it only affects the population above 67 years (SD not reported). The additive method was used to estimate disutility in subjects experiencing lifetime complications and vitamin D deficiency (25).

Utility estimates used in analysis are reported in the Table 1. It should be acknowledged that the methods used for utility estimation comes with considerable limitations. However, they are believed to be the best considering the level of available evidence. All utility estimates were tested in the threshold analyses to assess the impact they may have on the decision.

**Table 1:** Utility estimates used in the decision analytic model.

**Type of the parameter**	**Value**	**Evidence source**
Rickets		
Utility		
Mean (*SD*)	0·621 (0·18)	(21)
Assumed distribution	Beta (30·64; 18·70)	
Time in the state		
Mean (*SD*)	2 yr. (0·2)	Assumed SD; expert opinion
Assumed distribution	Gamma (100; 0·02)	
Vitamin D insufficiency		
Disutility		
Mean (*SD*)	0·2% (0·001)	(19)
Assumed distribution	Beta (958479·4; 478281120·8)	
Age at which disutility onsets		
Mean (*SD*)	67·04 yr. (6·70)	Assumed SD (19)
Assumed distribution	Normal (67·04; 6·70)	
Rickets related lifetime complications		
Disutility		
Mean (*SD*)	5% (0·005)	Expert opinion
Assumed distribution	Beta (38000;722000)	
Duration	Lifetime	Expert opinion

### Transition Probabilities Between Health States

The estimation of intervention effectiveness was based on two papers reporting the intervention implemented in Birmingham and the baseline rickets incidence rates in the target area, acquired after analysis of the observational dataset (2, 26). It was assumed that the effectiveness of vitamin D supplementation in reducing the incidence of rickets would be equivalent to that reported in Birmingham and this effect would be achieved by increasing supplement uptake rates in pregnant woman and children under 4 years of age, figures presented in Table 2 (2, 26).

**Table 2:** Estimated intervention effect on rickets and Healthy start supplement uptake.

**Parameter**	**Value**	**References**
Estimated risk change of having rickets (OR)^*^		(2)
Mean value (*SD*)	0·41 (0·34)	
Assigned distribution	Log-normal (0·41; 0·34)	
Baseline supplementation prevalence		(26)
Woman	41·00%	
Assigned distribution	Beta (6446·00; 9278·00)	
Children	9·64%	
Assigned distribution	Beta (4104·00; 42656·00)	
Change in the numbers of distributed supplements (%)		(26)
Woman supplements*^#^*		
Mean value (*SD*)	17 (0·17)	
Assigned distribution	Beta (2871·95; 14022·00)	
Children supplements*^#^*		
Mean value (*SD*)	20 (0·20)	
Assigned distribution	Beta (1999·97; 8000·00)	

* *OR – odds ratio, calculated from the reported figures*.

# *SD was not reported; hence it is assumed to be 10% of the reported mean*.

Scenario analysis where the time horizon is increased to lifetime required additional parameters associated with considerable uncertainty. There was no evidence on the frequency of life-long complications, included in this analysis hence expert estimates were used. The assumed mean is 10% (assumed SD 10% of the mean value) for all the population subgroups. In the PSA this parameter was assigned a beta distribution with parameters a = 8999·99 and b = 81000.

Vitamin D deficiency in people aged 77 years or greater, (*SD* = 8·84) is associated with increased mortality risk (3). To account to this, in the presented scenario analysis it was assumed that by the time the population reaches this age, vitamin D deficiency rates have reached the population baseline. Estimates of population vitamin D deficiency baseline was derived by pooling data from UK national surveys reported in SCAN report (Table S2) (3, 27, 28). The vitamin D deficiency-related mortality risk increase was estimated using figures acquired from Cochrane meta-analysis, RR for mortality = 1·05 (SE = 0·02); for patients aged 77 years or greater (3, 29). In the PSA the RR for mortality was assigned a Log-normal distribution with parameters a = 1·05, b = 0·02 and patient age was assigned a normal distribution with parameters a = 76·92, b = 8·84.

### Calculation of Resource Use and Costs

The model considered the following costs: rickets treatment, intervention, management of vitamin D deficiency and lifetime complications.

All cases of rickets diagnosed in Greater Manchester were identified and confirmed based on abnormal biochemistry (low 25OHD of <30nmol/l, high alkaline phosphatse and elevated parathyroid hormone) and confirmed radiological changes of rickets. Rickets treatment costs, till radiological signs of healing of rickets and normalization of biochemistry, were calculated from an observational dataset, using NHS tariffs 2016-2017 to derive unit prices (Table S3) (30). As these costs differed between population subgroups, group specific mean values were used. To account to the skewed data, a SD of 10% of the mean was assumed.

Intervention associated costs include costs of supplement acquisition (Table 3) and costs of intervention administration. It is assumed that administration costs also include the costs of a promotion campaign which was part of the campaign implemented in Birmingham and contributed, to an unknown extent, toward achieving the clinical effect (2, 26). The total price of the supplements per single patient is calculated as the price of supplements for children for the 4 year duration of the intervention and the price of supplements for pregnant woman for the duration of pregnancy.

**Table 3:** Prices of the vitamin D supplements (source: “Healthy Start”, 2018).

**Type of the supplement**	**Price per pack (£)**	**Days sufficient**	**Price per year/pregnancy (£)**
Woman's Healthy start supplement	0·74	56	3·57
Children's Healthy start supplement	1·52	56	9·90

To take into account the different ways in which the proposed program can be implemented, threshold analyses were conducted to inform an economically justifiable budget to cover costs of the promotion campaign, distribution of supplements and overheads. In the base case this is assumed to be £10,000 per year for the Central Manchester region.

In the scenario analysis where lifetime complications were assumed, costs of managing these and vitamin D deficiency-associated disutility are included. As there was no information identified, expert estimates were used (Table S3).

### Uncertainty Handling

Various uncertainties surrounding the decision problem were assessed following recommendations by Claxton (Supplementary Appendix), the results of these analyses are presented in the following sections (31).

### Role of the Funding Source

Thornton & Ross Pharmaceuticals, UK provided financial support only for collecting data on cost of management of rickets. No specific funding was used for data analysis, interpretation of results and writing of this manuscript.

## Results

### Observational Dataset Analysis

Demographic characteristics of children under 4 years of age in Central Manchester area are presented in Table S4 and for those with rickets in Table 4. Risk of rickets estimates based on diagnosis between Jan 2009 to Dec 2013 are presented in Table 5 (annual risk and incidence rate) and Table S5 (4-year risk). Analysis of this dataset revealed considerable differences in risks of rickets estimates and rickets management costs, between population subgroups Table 6 and Table S3. Children with dark skin tones were found to be the most at risk and their treatment costs were also the highest (£7,305 per patient), as they were more likely to suffer from rickets and vitamin D deficiency-associated complications such as hypocalcaemic seizures and dilated cardiomyopathy.

**Table 4:** Demographic characteristics of the incidence population (observational dataset).

**Parameter**	
Age at first admission (months)	
Mean (*SD*)	21·16 (22·42)
Sex *n* (%)	
Male	36 (63·16)
Female	21 (36·84)
Skin tone; *n* (%)	
Light	2 (3·51)
Medium	29 (50·88)
Dark	26 (45·61)

*No missing observations*.

**Table 5:** Estimated annual risks of having rickets and rickets incidence rates by skin tone (observational dataset).

	**General population**	**Children with medium and dark skin tones**	**Children with light skin**	**Children with medium skin tone**	**Children with dark skin tone**
n/N^*^	9·33/36,413	9·00/19,069	0·33/17,344	4·83/14,117	4·17/4,952
Per 100,000	25·63	47·19	1·92	34·23	84·14
children					

* *Calculated as average number of cases per year by number at risk which, was acquired from census data (14)*.

**Table 6:** Rickets management costs by population subgroup (observational dataset; 2016–2017 year price equivalent).

**Population subgroup**	**Mean (£)**	**Observed SD**
Children with light skin	1,750	48
Children with medium skin tone	2,385	3,759
Children with dark skin tone	7,305	16,208

### Base-Case Analysis

**Figure 2:**
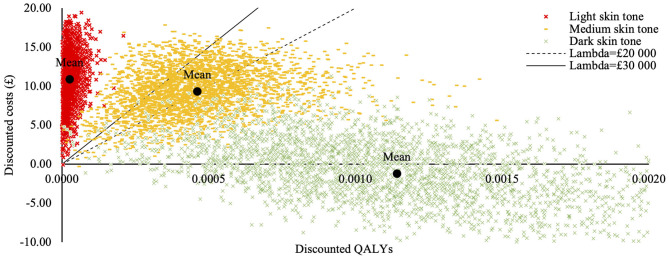
Cost-effectiveness acceptability plane for the homogenous population subgroups (base-case analysis). Cost effectiveness acceptability plane was generated in the probabilistic sensitivity analysis and illustrates differences in costs and treatment outcomes between current and alternative practices, taking parameter uncertainty into account. Each dot represents a random probabilistic estimate of the incremental costs and quality adjusted life years (QALYs). Cost-effectiveness probabilities presented in the base case analysis are calculated as a proportion of these samples below the threshold values. The mean price per QALY value for the subgroup consisting of children with dark skin tone is below the “x” axis meaning the intervention is cheaper than the current practice; the price per QALY estimates for the subgroup consisting of children with medium skin tone are scattered in the upper quadrant around the threshold lines, illustrating uncertainty of intervention's cost-effectiveness. The price per QALY estimates for the subgroup consisting of children with light skin tones are concentrated along the “y” axis, well above budget threshold lines, illustrating minimal health gains and considerably higher costs.

Deterministic base-case results are presented in Table 7 and suggest that intervention is cost saving while producing health benefits for children with a dark skin tone, cost-effective in children with a medium skin tone but not cost-effective in children with light skin tone. Probabilistic base-case analysis results are presented in Table 8 and the cost effectiveness plane is presented in Figure 2 (13).

**Table 7:** Base case analysis results (deterministic analysis).

**Description**	**Results**		**Incremental**		**ICER (£)**
	**Costs (£)**	**QALYs**	**Costs (£)**	**QALYs**	
Dark skin tone					
Comparator	21·46	3·334151			
Intervention	19·74	3·335339	−1·68	0·001188	Dominant
Medium skin tone					
Comparator	2·85	3·333224			
Intervention	12·14	3·333706	9·30	0·000482	19,295
Light skin tone					
Comparator	0·12	3·335305	-	-	-
Intervention	10·99	3·335332	10·91	0·000027	404,074

**Table 8:** Base case analysis results (probabilistic analysis).

**Description**	**Mean results**		**ICER (£)**			**Probability cost effective**	
	**Costs (£)**	**QALYs**	**Mean value**	**95% confidence interval**		**λ = £20,000 per QALY**	**λ = £30,000 per QALY**
				**Lower bound**	**Upper bound**		
Dark skin tone							
Comparator	£21·46	3·334138					
Intervention	£20·23	3·335279	Dominant	Dominant	Dominant	0·99	0·99
Medium skin tone							
Comparator	2·84	3·333228					
Intervention	12·17	3·333689	20,222	20,038	20,405	0·52	0·58
Light skin tone							
Comparator	0·12	3·335298					
Intervention	10·99	3·335323	423,340	417,256	£429,425	0·00	0·00

*Probabilistic analysis results are generated by assigning standard mathematical distributions reflecting model parameter uncertainty (presented throughout the text and in the Appendix) and then randomly drawing a sufficiently large sample (in this case 10,000 draws) of model outputs, which is then analyzed using standard statistical techniques (mean, interquartile range), ICER confidence intervals acquired using Jackknife method (Simulation Modeling and Analysis, 2000)*.

### Uncertainty Handling

**Figure 3:**
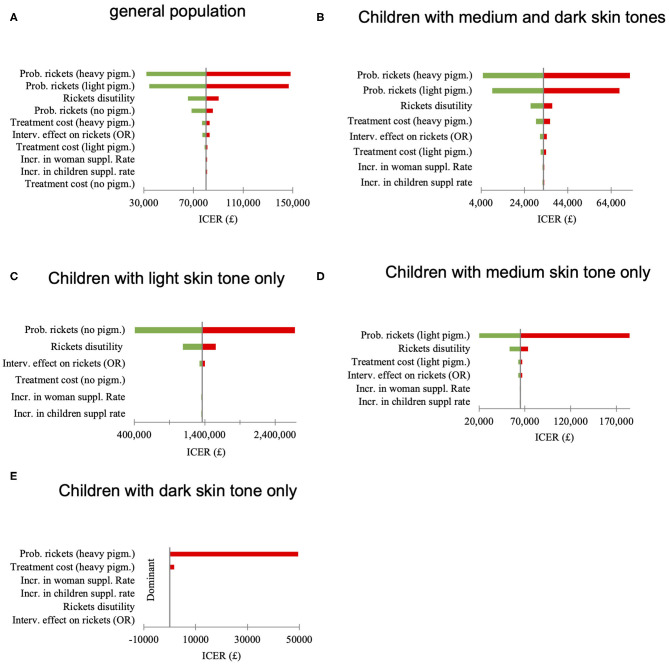
**(A–E)** Univariate sensitivity analysis, effect of the parameter variation between 2·5th and 97·5th quintile on the incremental cost-effectiveness ratio (ICER) value.

Univariate sensitivity analysis, where single parameters were changed between the lower and upper limits of their 95% confidence interval to test how this variation might influence the incremental cost-effectiveness ratio (ICER) value was undertaken. As shown in Figures 3A–E, the risk of having rickets could influence the adoption decision (i.e., whether the intervention was cost-effective) only in the medium skin tone subgroup. Other parameters were tested but did not alter the adoption decision in any population group.

Parameters elicited from experts, or where risk of bias was assessed to be high were tested in a threshold analysis (Table 9). In this analysis parameters were manipulated to identify the values at which the adoption decision changed. As in the previous analysis's adoption decision was the most certain for groups consisting of children with light and dark skin tones. In the medium skin tone group relatively insignificant changes in some of the parameter values (e.g., rickets duration, intervention costs without supplements, and change in supplementation rates) brought ICER values above the lower threshold value of £20,000. However, parameter values at which ICER exceeded the upper threshold value of 30,000 can be considered highly unlikely.

**Table 9:** Parameter threshold analysis to determine decision changing values for most uncertain parameters.

**Value at which ICER is equal or exceeds threshold value**							
**Description**	**Base case value**	**Light skin tone**		**Medium skin tone**		**Dark skin tone**	
		**ICER = 404,074**		**ICER = £19,295**		**Intervention dominates**	
Lambda (£)		20,000	30,000	20,000	30,000	20,000	30,000
Rickets duration (yr.)	2	40.45	26.96	1.93	1.28	<0	<0
Intervention costs (excl. supplements, £/yr.)	10,000	<0	<0	13,526	63,877	275,731	399,832
Intervention OR for rickets incidence	0·410	<0.001	<0.001	0.427	0.597	0.822	0.866
Increase in woman supplementation rates (%)	17	<0	<0	20	54	>100	>100
Increase in children supplementation rates	20·0	<0	<0	21	33	79	>100

*Results of the threshold analysis testing baseline QoL presented in Supplementary Appendix*.

Structural uncertainty around life-long complications was assessed in scenario analysis, where the modeled time horizon was extended to lifetime. The results presented in Table 10 show that the ICERs are reduced compared to the base case model with a 4-year time horizon but not to a point at which the intervention became cost-effective in a light skin tone population.

**Table 10:** Scenario analysis assuming lifelong complications.

	**Deterministic ICER (£)**	
	**Base case value**	**Scenario estimate**
Children with light skin tone	404,074	351,613
Children with medium skin tone	19,295	16,996
Children with dark skin tone	Intervention dominates	Intervention dominates

## Discussion

To the best of our knowledge, this analysis is the first cost-effectiveness study of a vitamin D supplementation-based rickets prevention programme in pregnant women and children <4 years of age to prevent rickets in children. An analysis conducted by York Health Economics Consortium concerning vitamin D was a cost-consequence analysis and is of limited comparability (32). The study conducted in Birmingham investigated the clinical impact of supplementation rather than cost-effectiveness (2, 26).

Our study shows that such a programme dominated standard care (increasing health outcomes while reducing costs) in participants who have a dark skin tone but is not cost-effective in participants whose skin tone is light. In participants with a medium skin tone the deterministic ICER is £19,295 per QALY; PSA: £20,222 per QALY crossing the willingness to pay threshold adopted by NICE of £20,000 per QALY but still below the upper value of £30,000 per QALY.

In our analysis the annual rickets incidence in general population was found to be 26 cases per 100,000 children under 4 years of age, significantly lower than that reported in Birmingham (120 cases per 100,000 children under 5 years of age) (2). We believe that the difference is due to geographical, socio-economic or methodological differences between the two studies and that our results are applicable to locations where the annual incidence rate of rickets is similar or higher.

Univariate sensitivity analyses showed that rickets prevalence was the main driver of cost-effectiveness. Baseline rickets prevalence is the highest in dark skin tone participants and lowest in light skin tone participants as evidence from our and other studies suggests (3). Our results extend this evidence and suggest a relationship exists between skin pigmentation levels and the cost effectiveness of a vitamin D supplementation programme to prevent rickets in children.

Another univariate sensitivity analysis was conducted to address uncertainty around baseline HRQoL estimate (0·9607) which was varied between the quartiles for the difference between HRQoL for children in perfect health (1·000) and children with rickets (0·621) (Table S6). The decision remained unchanged for participants with dark and participants with a light skin tone. However, for participants with a medium skin tone the decision would change between the 25th quartile (0·905) ICER = £23,192 and the 50th quartile (0·811) ICER = £34,701 if threshold of £30,000 per QALY is assumed and between the 100th (1·000) and 75th (0·905) quartiles if threshold of £20,000 per QALY is assumed.

The base case analysis results were estimated assuming a four-year time horizon and zero probability of lifetime complications. It is known that rickets is associated with significant effects on health in the short-term. It is also believed that rickets in some cases has longer-term (lifetime) effects on health. When these are included (scenario analysis) the level of uncertainty in the results increases significantly because parameters associated with longer-term complications are based on expert opinion. If lifetime complications do exist at the levels our experts believe, and if other health benefits to pregnant women and children are also taken into consideration (33–35), then the ICER will be lower than estimated in the base case analysis, thereby increasing the cost-effectiveness of the intervention.

In summary, our study demonstrates that population-based Healthy Start Vitamin D Supplementation Programme in pregnant women and children under 4 years of age aimed to lower rickets incidence rates in children is clinically effective and cost-saving in participants with a dark skin tone, cost effective in participants with a medium skin tone (assuming threshold of £30,000 per QALY) but not cost-effective in participants with a light skin tone. We believe that results of this study will help to guide policy makers in allocating resources and implementing vitamin D supplementation programmes to prevent rickets in children.

## Data Availability Statement

All datasets presented in this study are included in the article/Supplementary Material.

## Ethics Statement

Ethical review and approval was not required for the study on human participants in accordance with the local legislation and institutional requirements. Written informed consent from the participants' legal guardian/next of kin was not required to participate in this study in accordance with the national legislation and the institutional requirements.

## Author Contributions

VF, ZM, and RP: study design and manuscript preparation. VF, FJ, ZM, and RP: study investigator. FJ, AD, IZ, and RP: collection and assembly of data. VF, AR, and MS: data analysis. All authors: data interpretation, manuscript review and revisions, and final approval of manuscript.

## Conflict of Interest

VF: Affiliated to University of Sheffield when he undertook this project and was undertaking internship at Dolon, UK. VF confirms that he conducted this study in the absence of any commercial or financial relationships with Dolon Ltd that could be construed as a potential conflict of interest. RP: Received financial support for this study for collecting data on cost of management of rickets from Thornton & Ross Pharmaceuticals, UK. The remaining authors declare that the research was conducted in the absence of any commercial or financial relationships that could be construed as a potential conflict of interest.
