# Supplementary material for: Cost-Effectiveness of Vitamin D Supplementation in Pregnant Woman and Young Children in Preventing Rickets: A Modeling Study
Source: Front Public Health. 2020 Sep 4;8:439. doi: 10.3389/fpubh.2020.00439 (PMC7498641; doi:10.3389/fpubh.2020.00439)
Supplement: Supplementary file 1 [file Data_Sheet_1.docx]

SUPPLEMENTARY APPENDIX

**Cost-effectiveness of Vitamin D supplementation in pregnant woman and young children in preventing rickets in children**

Vilius Floreskul^1^, Fatema Z Juma^2^, Anjali B Daniel^3^, Imran Zamir^4^, Andrew Rawdin^5^, Matthew Stevenson^5^ Dr. Zulf Mughal^2,3^ Dr. Raja Padidela^2,3^

**Author affiliations**

1. Dolon Ltd, UK
2. Faculty of Biology, Medicine and Health, University of Manchester, Manchester Academic Health Science Centre, Manchester, UK
3. Royal Manchester Children’s Hospital, Manchester University NHS Foundation Trust
4. North Manchester General Hospital, UK
5. School of Health and Related Research, The University of Sheffield, UK

**Corresponding author:**

Dr. Raja Padidela

Royal Manchester Children’s Hospital

Manchester University NHS Foundation Trust

Oxford Road, Manchester M13 9WL

Tel: 0161 7011628

Fax: 0161 7011631

Email: [Raja.Padidela@mft.nhs.uk](mailto:Raja.Padidela@mft.nhs.uk)

**Discounting**

Following National Institute’s for Health and Care Excellence reference case health and costs in this analysis were discounted using standard 3·5% discount rate.^1^

**Systematic literature searches targeting Vitamin D deficiency and** **rickets related disutility**

With regards to rickets and vitamin D insufficiency-related disutility, two separate systematic literature searches were conducted. The literature search for QoL data is rather different from that of the efficacy data and whilst there is a range of guidelines on conducting systematic literature searches for efficacy data, there are no formal ones for QoL data.^2^ QoL is rarely reported amongst primary or secondary study outcomes and hence is not well indexed. In addition, it is difficult to conceptualise QoL search queries, using a standard population-intervention-comparator-outcome-study type approach, because QoL data can come from a range of different studies, including observational studies which might not have a comparator or intervention. This is especially the case in poorly researched areas like rickets or vitamin D insufficiency. These reasons led to the decision to design low specificity and high sensitivity search queries in order not to miss any relevant information.

The search queries included filters designed, validated and currently used by the Patient Reported Outcome Measurement (PROM) Group at University of Oxford and a MeSH terms for rickets and 25OH(D) (detailed search terms are available below).^3^ Both search queries were in line with inclusion and exclusion criteria defined in advance. The search for vitamin D deficiency-related QoL data was conducted on 03/08/2018 and 12/07/2018 for rickets in the Medline database using PubMed interface. In addition to that, following the triangulation approach, listed databases and literature lists of identified publications^4,5^ were manually reviewed:

- Cost-effectiveness Analysis Registry;^6^
- Centre for Reviews and Dissemination databases;^7^
- Health Economics and Evaluation database;^4^
- Research Papers in Economics;^8^
- Patient-reported outcome and quality of life instruments database.^9^

The search results are presented in Figure-S1.

The Rickets-targeted search resulted in 756 citations, of which only one was found to be relevant. No relevant studies were identified manually searching the mentioned databases. The search query was constructed in a way it would not exclude meta-analyses, however no such studies were identified. After a review of 34 full texts it was found that 25 papers were concerned with a similar disease area but did not assess quality of life in rickets and thus were excluded, six were conference proceeding or articles for which the full text could not be retrieved, and one was in the French language and was excluded. Of the remaining two studies one paper used an unvalidated instrument and thus was also excluded leaving a single study, which was assessed against criteria outlined in TSD 9.^2,10^ Reference mining, where reference lists of these papers were reviewed and assessed, did not result in identification of any other relevant studies.

With regards to the search targeted to vitamin D insufficiency related QoL loss, 860 citations were identified. The reference mining resulted in identification of one meta-analysis, which assessed QoL in relation to vitamin D supplement use status, but not to serum 25OH(D) concentration; which was the outcome of interest. The reference list of the identified meta-analysis was also reviewed, however no relevant studies assessing QoL based on serum 25OH(D) concentration levels were identified. No relevant studies were identified during the databases search. Overall the search resulted in identification of two studies, one conducted in China, and the other in Canada.^11,12^ The Canadian study was chosen to inform the model, as its population was assumed to be more homogenous with the target population in this analysis. Its quality was assessed against criteria outlined in Table-S1^2^

Figure S1. Summary of the systematic literature search results (PRISMA Flow Diagrams)

| Rickets related disutility | Vitamin D insufficiency related disutility |
| --- | --- |
| Citations identified  n=756  Citations identified by experts  n=1  Title and abstract screening  Rejected articles  n=722  Selected for full-text review and reference mining  n=35  Full electronic paper retrieval, review and reference mining  Identified during the reference mining  n=0  Relevant articles  n=1  Articles rejected after full-text review  QoL not assessed; n=25  Full text not available; n=6  Non-English study; n=1  Use of non-validated measure; n=1  High risk of bias; n=1 | Citations identified  n=860  Title and abstract screening  Rejected articles  n=821  Selected for full-text review and reference mining  n=39  Full electronic paper retrieval, review and reference mining  Identified during the reference mining  n=1  Relevant articles  n=1  Articles rejected after full-text review  QoL not assessed; n=34  Full text not available; n=2  Female only population; n=1  Inappropriate outcomes; n=1  Inappropriate context; n=1 |
| Study inclusion criteria: |  |
| - Vitamin D deficiency rickets in children without comorbidities; - Use of validated QoL measure. | - Vitamin D deficiency without comorbidities; - Use of validated QoL measure. |
| Study exclusion criteria: |  |
| - Comorbid populations, non-vitamin D deficiency related rickets; - Use of non-validated QoL measures; - Non-human or case studies. | - Comorbid populations; - Serum 25OH(D) levels not assessed; - Use of non-validated QoL measures; - Non-human or case studies. |

The study for QoL in rickets was assessed to have a high risk of bias, and hence was latter replaced by a higher quality study published after the systematic literature search, identified by the field experts.^13^

With regards to vitamin D insufficiency related QoL, the identified study was assessed as being at low risk of bias. QoL in this study was measured using EQ-5D-5L and converted to utility values using a US valuation study.^14^ As individual sub-scores required for conversion between two scales were unavailable, it was assumed this disutility, with reference to the baseline, is proportionate between the EQ-5D-5L and EQ-5D-3L required by NICE reference case. Vitamin D insufficiency related disutility is only considered in the scenario analysis, when the time horizon is lifetime, its impact is negligible and only applies when the population reaches the age of 67.04 years (SD = 6.70; assumed to be equal to 10% of the mean value), hence was assumed to be justifiable. The additive method was used to estimate disutility for populations which experienced lifetime complications and vitamin D insufficiency.^15^

**Search query for rickets related QoL**

("Rickets"[Mesh]) AND ((HR-PRO[tiab] OR HRPRO[tiab] OR HRQL[tiab] OR HRQoL[tiab] OR QL[tiab] OR QoL[tiab] OR quality of life[tw] OR life quality[tw] OR health index*[tiab] OR health indices[tiab] OR health profile*[tiab] OR health status[tw] OR ((patient[tiab] OR self[tiab] OR child[tiab] OR parent[tiab] OR carer[tiab] OR proxy[tiab]) AND ((report[tiab] OR reported[tiab] OR reporting[tiab]) OR (rated[tiab] OR rating[tiab] OR ratings[tiab]) OR based[tiab] OR (assessed[tiab] OR assessment[tiab] OR assessments[tiab]))) OR ((disability[tiab] OR function[tiab] OR functional[tiab] OR functions[tiab] OR subjective[tiab] OR utility[tiab] OR utilities[tiab] OR wellbeing[tiab] OR well being[tiab]) AND (index[tiab] OR indices[tiab] OR instrument[tiab] OR instruments[tiab] OR measure[tiab] OR measures[tiab] OR questionnaire[tiab] OR questionnaires[tiab] OR profile[tiab] OR profiles[tiab] OR scale[tiab] OR scales[tiab] OR score[tiab] OR scores[tiab] OR status[tiab] OR survey[tiab] OR surveys[tiab]))))

**Search query for vitamin D insufficiency related QoL**

(("Calcitriol"[Mesh]) OR "25-Hydroxyvitamin D 2"[Mesh]) AND ((((HR-PRO[tiab] OR HRPRO[tiab] OR HRQL[tiab] OR HRQoL[tiab] OR QL[tiab] OR QoL[tiab] OR quality of life[tw] OR life quality[tw] OR health index*[tiab] OR health indices[tiab] OR health profile*[tiab] OR health status[tw] OR ((patient[tiab] OR self[tiab] OR child[tiab] OR parent[tiab] OR carer[tiab] OR proxy[tiab]) AND ((report[tiab] OR reported[tiab] OR reporting[tiab]) OR (rated[tiab] OR rating[tiab] OR ratings[tiab]) OR based[tiab] OR (assessed[tiab] OR assessment[tiab] OR assessments[tiab]))) OR ((disability[tiab] OR function[tiab] OR functional[tiab] OR functions[tiab] OR subjective[tiab] OR utility[tiab] OR utilities[tiab] OR wellbeing[tiab] OR well being[tiab]) AND (index[tiab] OR indices[tiab] OR instrument[tiab] OR instruments[tiab] OR measure[tiab] OR measures[tiab] OR questionnaire[tiab] OR questionnaires[tiab] OR profile[tiab] OR profiles[tiab] OR scale[tiab] OR scales[tiab] OR score[tiab] OR scores[tiab] OR status[tiab] OR survey[tiab] OR surveys[tiab]))))))

**Quality assessment of the studies considered for utility estimation**

Table S1. Quality assessment of the studies identified in the systematic literature review and identified by clinical experts.

| **Rickets related QoL**^10^ | **Rickets related QoL**^13^ | **Vitamin D deficiency related QoL**^12^ |
| --- | --- | --- |
| Sample size |  |  |
| 36, 0-3-year-old children with vitamin D deficiency rickets. | 133 clinical experts | 2119 adult people. |
| Respondent selection and recruitment |  |  |
| Recruitment procedure unclear, high probability that the study population is not homogenous with the target population in the conducted cost-effectiveness analysis. | Recruitment procedure was organised through the professional societies of relevant specialisation clinicians in the EU and UK | Respondents that took part in an health promotion programme in Canada, likely not representative to the overall UK population, however similar to UK in terms of income levels, exposure to the sunlight, culture, etc. |
| Inclusion/ exclusion criteria |  |  |
| Not reported | All experts | Not reported, likely none. |
| Response rates |  |  |
| Not reported | 27% - possible impact of the selection bias | 84·7% at 6 months  35·9% at 12 months, likely does not have effect on QoL estimates, however the procedures are not explicitly reported |
| Loss to follow-up |  |  |
| Not reported | Same as above | Same as above |
| Missing data |  |  |
| Not reported | Not reported | Not reported |
| Other concerns |  |  |
| Study is poorly reported and was conducted in Armenia, hence the population is not homogenous with the target population in our study. | No concerns | No other concerns |
| Appropriateness of the measure |  |  |
| QUALIN, a French validated QoL measure specific for children, study presents unevaluated values from the descriptive system ^16^. Validated in children, psychometric properties in this disease area were not assessed. | Study adopted validated instruments and appears to be conducted to a high standard | EQ-5D-5L, Suitable for target population, psychometric properties in vitamin D deficiency are not assessed. |
| Overall risk of bias |  |  |
| High risk of bias | Low-medium risk of bias | Low risk of bias |
| Inclusion in the analysis | | |
| Excluded from the analysis | Included in the analysis | Included in the analysis |

**Vitamin D insufficiency prevalence**

Table S2. Baseline vitamin D insufficiency prevalence.

| **Population subgroup** | **Parameter** | **Evidence category/ source** |
| --- | --- | --- |
| Probability of vitamin D insufficiency adjusted by age and sex | |  |
| Man (11-65+ years old) |  | ^17^ |
| n/N | 210 / 754 |  |
| Assumed distribution | Beta(210;754) |  |
| Woman (11-65+ years old) |  |  |
| n/N | 276 / 942 |  |
| Assumed distribution | Beta(276;942) |  |
| Odds ratio to adjust by skin pigmentation level | | ^18^ |
| Light skin |  |  |
| Mean OR (SE) | 0·84 (0·06) |  |
| Assumed distribution | Log-Normal(0·83; 0·05) |  |
| Medium skin tone |  |  |
| Mean OR (SE) | 7·12 (0·16) |  |
| Assumed distribution | Log-Normal(7·12; 0·16) |  |
| Dark skin tone |  |  |
| Mean OR (SE) | 3·72 (0·23) |  |
| Assumed distribution | Log-Normal(3·73;0·24) |  |

**Cost of management of rickets**

Table S3. Rickets treatment prices by population subgroup (2016/17year price equivalent).

| **Population subgroup** | **Mean (£)** | **Assumed SD** | **Assigned distribution** | **Source** |
| --- | --- | --- | --- | --- |
| Rickets managemet costs (per case) |  |  |  |  |
| Children with light skin tone | 1750 | 17·50 | Gamma(100;17·50) | Observational dataset |
| Children with medium skin tone | 2385 | 23·82 | Gamma(100;23·82) | Observational dataset |
| Children with dark skin tone | 7305 | 73·05 | Gamma(100;73·05) | Observational dataset |
| Lifetime complication management (per year) | 100 | 10·00 | Gamma (100;1) | Expert opinion |
| Vitamin D insufficiency related disutility management | 100 | 10·00 | Gamma (100;1) | Expert opinion |

**Uncertainty handling**

The presented decision problem deals with uncertainty related to: parameter estimates and applied methods; variability between patients with different characteristics; uncertainty around model structure/ assumptions. Parameter uncertainty was addressed by systematic use of information; in addition to that, highly uncertain parameters were tested in the univariate sensitivity and threshold analyses; variation in the estimated means – by assigning assumed distributions and running probabilistic sensitivity analyses (PSAs); methodological uncertainty – by following NICE reference case; patient heterogeneity – by conducting separate analyses for population groups by skin tones.^1^ Structural uncertainty is the most difficult to formally assess, hence analysis results have to be interpreted in consideration of it. A degree of this uncertainty surrounds the intervention and its effectiveness and would be highly dependent on implementation technicalities. Other areas concerned with this uncertainty are the inclusion of life-long complications, model timeline and conditions associated with vitamin D insufficiency not included in the model. The impact of these aspects is discussed in the discussion section.

**Population characteristics**

Table S4. 0-4-year-old children distribution by skin tone and sex in Central Manchester.

|  | **Children with light skin tone** | **Children with medium skin tone** | **Children with dark skin tone** | **Source** |
| --- | --- | --- | --- | --- |
| Male (N=18,615) |  |  |  |  |
| n | 8,964 | 7,035 | 2,616 | Census ^19^ |
| Assigned probability | 1. minus the joint probability of being in any other homogenous group | Beta(7,035; 29,378) | Beta(2,616; 33,797) | Assumption |
| Female (N=17,798) |  |  |  |  |
| n | 8,380 | 7,082 | 2,336 | Census ^19^ |
| Assigned probability | Beta(8,380; 28,033) | Beta(7,082; 29,331) | Beta(2,336; 34,077) | Assumption |

Table S5. Estimated 4-year risks of having rickets (Observational dataset, Central Manchester population).

|  | **Children with light skin** | **Children with medium skin tone** | **Children with dark skin tone** | **Source** |
| --- | --- | --- | --- | --- |
| Male |  |  |  |  |
| n/N | 1·3 / 17,344 | 13·6 / 7,035 | 10·7 / 2,616 | Obs. dataset/Census ^19^ |
| Assumed distribution | Beta(1·3; 17,342·7)^!^ | Beta(13·6; 7,021·4) | Beta(10·7; 2,605·3) | Assumption |
| Female |  |  |  |  |
| n/N | 1·3 / 17,344^!^ | 8·0 / 7,082 | 6·0 / 2·336 | Obs. dataset/Census ^19^ |
| Assumed distribution | Beta(1·3; 17,342·7)^!^ | Beta(8·0; 7,074) | Beta(6·0; 2,330) | Assumption |

*^!^*No rickets cases in females having light skin were observed, as there were only 3 cases in their male counterparts, it was assumed that such results are by chance and hence male and female risks were pooled.

**Baseline QoL impact on estimated ICER values**

Table S6. Baseline QoL impact on estimated ICER values (£).

| Tested value | Light skin tone only | Medium skin tone only | Dark skin tone only |
| --- | --- | --- | --- |
| 0·9607 (base case) | 404,074 | 19,295 | Intervention dominates |
| 1·00 | 363,667 | 17,383 | Intervention dominates |
| 0·905 | 474,348 | 23,192 | Intervention dominates |
| 0·811 | 727,333 | 34,701 | Intervention dominates |
| 0·716 | 1,363,750 | 69,403 | Intervention dominates |

Tested values selected as 100^th^, 75^th^, 50^th^ 25^th^ quartiles of the difference between QoL in full health (utility = 1·000) and QoL with rickets (utility = 0·621)

**Literature**

1 Guide to the methods of technology appraisal. National Institute for Health and Care Excellence. <https://www.nice.org.uk/process/pmg9/resources/guide-to-the-methods-of-technology-appraisal-2013-pdf-2007975843781>. Published 2013. Accessed Aug, 2020

2 Papaioannou D, Brazier J, Paisley S. Systematic searching and selection of health state utility values from the literature. *Value Heal* 2013. DOI:10.1016/j.jval.2013.02.017.

3 Terwee CB, Jansma EP, Riphagen II, De Vet HCW. Development of a methodological PubMed search filter for finding studies on measurement properties of measurement instruments. *Qual Life Res* 2009. DOI:10.1007/s11136-009-9528-5.

4 Wiley Interscience. HEED: Health Economics Evaluation Database. 2017.

5 Centre for Reviews and Dissemination. CRD Database. 2018.

6 Cost effectiveness analysis registry. Search the CEA Registry. http://healtheconomics.tuftsmedicalcenter.org/cear4/SearchingtheCEARegistry/SearchtheCEARegistry.aspx (accessed Aug, 2020).

7 Centre for Reviews and Dissemination. NIHR Centre for Reviews and Dissemination - CRD Database. https://www.crd.york.ac.uk/CRDWeb/ (accessed Aug, 2020).

8 Research Papers in Economics. RePEc: Research Papers in Economics. http://repec.org/ (accessed Aug, 2020).

9 MAPI Institute. PROVIDE^TM^. https://eprovide.mapi-trust.org/subscriptions (accessed Aug, 2020).

10 Dunamalyan R, Mardiyan M, Danielyan L, Mkrtchyan S, Chopikyan A. QUALITY OF LIFE OF CHILDREN WITH RICKETS IN YOUNG AGE IN ARMENIA. *Georgian Med News* 2017; : 60—64.

11 Feng X, Guo T, Wang Y, *et al.* The vitamin D status and its effects on life quality among the elderly in Jinan, China. *Arch Gerontol Geriatr* 2016. DOI:10.1016/j.archger.2015.09.002.

12 Ekwaru JP, Ohinmaa A, Veugelers PJ. The effectiveness of a preventive health program and vitamin D status in improving health-related quality of life of older Canadians. *Qual Life Res* 2016. DOI:10.1007/s11136-015-1103-7.

13 Aguiar M, Andronis L, Pallan M, Högler W, Emma F. Micronutrient deficiencies and health-related quality of life: the case of children with vitamin D deficiency. *Public Health Nutr* 2019.

14 Herdman M, Gudex C, Lloyd A, *et al.* Development and preliminary testing of the new five-level version of EQ-5D (EQ-5D-5L). *Qual Life Res* 2011. DOI:10.1007/s11136-011-9903-x.

15 Ara R, Wailoo A. NICE DSU Technical Support Document 12: The Use of Health State Utility Values in Decision Models. 2014.

16 Manificat S, Dazord A, Langue J, *et al.* Evaluation de la qualité de vie du nourrisson et du très jeune enfant: Validation d’un questionnaire. Étude multicentrique européenne. *Arch Pédiatrie* 2000; **7**: 605–14.

17 Lennox A, Olson a, Gay C. National diet and nutrition survey. *Headl results from Years* 2011.

18 Health Survey. Health Survey for England 2015 Adult Overweight and Obesity. 2016.

19 Office for National Statistics. Nomis - Official Labour Market Statistics. https://www.nomisweb.co.uk/ (accessed Aug, 2020).
